# Attitudes toward harm reduction and low-threshold healthcare during the COVID-19 pandemic: qualitative interviews with people who use drugs in rural southern Illinois

**DOI:** 10.1186/s12954-022-00710-9

**Published:** 2022-11-19

**Authors:** Alex Rains, Mary York, Rebecca Bolinski, Jerel Ezell, Lawrence J. Ouellet, Wiley D. Jenkins, Mai T. Pho

**Affiliations:** 1 Department of Medicine, University of Chicago, Chicago, IL USA; 2 Department of Sociology, Southern Illinois University, Carbondale, IL USA; 3 Africana Studies and Research Center, Cornell University, Ithaca, NY USA; 4 Center for Cultural Humility, Cornell University, Ithaca, NY USA; 5 Division of Epidemiology and Biostatistics, School of Public Health, University of Illinois Chicago, Chicago, IL USA; 6 Department of Epidemiology and Biostatistics, SIU School of Medicine, Springfield, IL USA

**Keywords:** COVID-19, People who use drugs, Harm reduction, Access to health services, Qualitative analysis, Secondary distribution, Low-threshold healthcare, Rural

## Abstract

**Background:**

Chronic health conditions associated with long-term drug use may pose additional risks to people who use drugs (PWUD) when coupled with COVID-19 infection. Despite this, PWUD, especially those living in rural areas, may be less likely to seek out health services. Previous research has highlighted the increased disease burden of COVID-19 among PWUD. Our manuscript supplements this literature by exploring unique attitudes of PWUD living in rural areas toward the pandemic, COVID-19 vaccination, and the role of harm reduction (HR) organizations in raising health awareness among PWUD.

**Methods:**

Semi-structured interviews were conducted with 20 PWUD living in rural southern Illinois. Audio recordings were professionally transcribed. A preliminary codebook was created based on interview domains. Two trained coders conducted iterative coding of the transcripts, and new codes were added through line-by-line coding and thematic grouping.

**Results:**

Twenty participants (45% female, mean age of 38) completed interviews between June and November 2021. Participants reported negative impacts of the pandemic on mental health, financial wellbeing, and drug quality. However, the health impacts of COVID-19 were often described as less concerning than its impacts on these other aspects of life. Many expressed doubt in the severity of COVID-19 infection. Among the 16 unvaccinated participants who reported receiving most of their information from the internet or word of mouth, uncertainty about vaccine contents and distrust of healthcare and government institutions engendered wariness of the vaccination. Distrust of healthcare providers was related to past stigmatization and judgement, but did not extend to the local HR organization, which was unanimously endorsed as a positive institution. Among participants who did not access services directly from the HR organization, secondary distribution of HR supplies by other PWUD was a universally cited form of health maintenance. Participants expressed interest in low-threshold healthcare, including COVID-19 vaccination, should it be offered in the local HR organization’s office and mobile units.

**Conclusion:**

COVID-19 and related public health measures have affected this community in numerous ways. Integrating healthcare services into harm reduction infrastructures and mobilizing secondary distributors of supplies may promote greater engagement with vaccination programs and other healthcare services.

***Trial number*:**

NCT04427202.

## Introduction

Since 1999, nearly 1 million people have died from a drug overdose [1]. In particular, people who use drugs (PWUD) living in rural environments may be disproportionately impacted by comorbidities associated with drug use, including mental illness, HIV, hepatitis C and other chronic diseases [2–4]. The COVID-19 pandemic has exacerbated many negative health outcomes, and may pose an increased risk to PWUD with respiratory and cardiovascular conditions that are related to long-term drug use [5]. Furthermore, the pandemic has been accompanied by spikes in overdose deaths, with mortality in 2020 totaling over 93,000, a substantial increase over the 72,000 deaths reported in 2019 [6]. Public health mitigations such as isolation and lock downs may also impact the mental health of PWUD by increasing loneliness, depression, and suicidal ideation [7]. Finally, pandemic-related shutdowns may impact the availability of substance use treatment and harm reduction services important to health maintenance among PWUD, including sterile syringe programs, take-home naloxone (Narcan), safe consumption sites, and medications for opioid use disorder (MOUD) [8, 9]. The disparities facing PWUD as a result of the pandemic may be compounded for those living in rural areas, which may be underresourced and socially isolated, creating a unique risk environment for these individuals.

Public distribution of COVID-19 vaccines began in early 2021, presenting new opportunities for—and challenges to—addressing the pandemic. Despite widespread availability, vaccine hesitancy poses a major barrier to broad uptake of the intervention [10]. Concerns about potential safety and misinformation around vaccine contents are commonly cited reasons for reluctance to get vaccinated. These concerns may be compounded by skepticism among certain populations toward mainstream healthcare providers which discourages vaccination [11]. People in rural communities demonstrate greater healthcare avoidance than people in urban areas, with physician mistrust and poor provider rapport contributing to disengagement, which may translate to vaccine hesitancy [12, 13]. PWUD in particular may choose to avoid healthcare settings due to stigmatization by medical providers [14]. Given the increased risk of severe COVID-19 disease among PWUD with drug use-related respiratory and cardiovascular conditions [5] and the likelihood of future pandemics that may call for swift vaccine interventions, it is important to understand potential barriers to vaccination in this population as a means of improving both the health of rural PWUD and the overall public health impact of vaccine programs.

Given that PWUD often avoid traditional healthcare settings due to combinations of mistrust, stigmatization and fear of being reported to authorities including law enforcement and child protective services, there is a need for alternative approaches. These approaches include integration of clinical services through peer-oriented harm reduction services and the concentrated expansion of those services through secondary distribution by PWUD of harm reduction supplies (e.g., syringes, naloxone) and health-related information to those who do not formally participate in these services [15]. These strategies can provide non-traditional access to healthcare, whereby clinical services are provided at community-level organizations trusted by PWUD rather than restricted to institutions that many PWUD avoid.

This study was designed to explore the impact of the COVID-19 pandemic on the overall wellbeing and drug use patterns of PWUD in the 16 southernmost counties in Illinois. These counties comprise part of the Delta Regional Authority, a federally designated area with high rates of unemployment and lower per capita income compared to other US counties [16]. This primarily rural region of Illinois is remarkable for characteristics related to its pandemic response as well as drug use characteristics. COVID-19 rates totaled over 40,000 cases per 100,000 people in 13 of the 16 counties studied [17], though vaccination rates in all 16 counties are lower than the state average [18]. In addition, several counties in this region are characterized by high rates of overdose deaths and limited access to primary care and mental health providers [19]. In spite of recent increases in rural drug use and associated fatality rates, this region has the fewest MAT facilities in Illinois [20] and only one harm reduction organization, which offers services through their brick-and-mortar location and mobile van units [21].

Given these regional characteristics, we aimed to explore attitudes of PWUD living in these counties toward the pandemic and COVID-19 vaccination efforts. Given the strain on PWUD-related service delivery presented by COVID-19, we explored community strategies for maintaining health and safety, such as secondary distribution of harm reduction supplies, especially given the strain on infrastructure that the COVID-19 pandemic presented. We also investigated responses to healthcare and vaccination efforts. Finally, we explored participants’ perceptions of harm reduction organization-based medical care, whereby PWUD can obtain traditional health services in a trusted environment.

## Methods

### Study design

This analysis was a part of the Ending Transmission of HIV, Hepatitis C, Sexually Transmitted Infectious and Overdose in rural communities of people who inject drugs (ETHIC) study, the Illinois site of the federally funded Rural Opioid Initiative [22]. Interview guides were developed using the Consolidated Framework for Implementation Research (CFIR) to explore the barriers and facilitators experienced by PWUD seeking to obtain harm reduction services [23]. We focused on the overarching CFIR domain of *Outer Setting* to evaluate external influences on harm reduction service delivery and patient needs. Questions around the impact of COVID-19, beliefs about healthcare and harm reduction services, and community-level characteristics were incorporated in this domain.

Participants’ experiences with and perspectives on drug use, harm reduction, healthcare and social service utilization, and the impact of COVID-19 on these factors, were examined using open-ended interview questions with optional probes that explored specific aspects of a question. For example, one interview question asked, *“How, if at all, has your drug use been impacted by COVID-19?”* and a probe of this question asked, *“Have you noticed any changes to the quality or properties of the drugs? [If yes] what were these changes?”* Interviewers reviewed the interview guide together prior to the start of the interview process and piloted the interviews with two PWUD in order to standardize the approach.

### Data collection

Interviews took place between June and November of 2021 in the following Illinois counties: Alexander, Franklin, Gallatin, Hamilton, Hardin, Jackson, Johnson, Massac, Perry, Pope, Pulaski, Randolph, Saline, Union, White, and Williamson. Interview participants were recruited face-to-face and over the phone by a local harm reduction agency using purposive and respondent-driven sampling [24]. Clients of the harm reduction agency were notified via text and during supply drop-offs that a study into the pandemic was being conducted and that they were able to opt in, with assurances made that no identifiable information discussed in the interview would be shared with staff at the agency. In order to minimize potentially biased recruitment of only those PWUD who engaged with the harm reduction agency most, interviewees were asked to refer other PWUD—both those who received services from the agency and those who did not—to participate in the study as well. Inclusion criteria included residence in the study counties, past 30-day injection of any illicit drugs or non-medical use of opioids through any route, English-speaking, and being at least 15 years old. All procedures were approved by the Institutional Review Board at the University of Chicago. The interview process, role of the interviewer in the ETHIC team, and goals of the present research were described and consent was obtained from each participant prior to their interview. Interviewees were paid $40 for participating in the interview and $20 for each eligible peer they referred for an interview.

Data were collected through semi-structured, audio-recorded narrative interviews conducted by ETHIC team members from the University of Chicago and Southern Illinois University trained in qualitative methods. Some interviews took place in person at a local harm reduction organization, including in its mobile delivery unit, and some over the phone or video call, depending on COVID-19-related risks. Interviews were conducted privately. Once the interview concluded, participants were able to obtain supplies and services from staff at the harm reduction organization.

### Data analysis

Interviews averaged roughly 45 min per participant. A numeric ID was generated for each participant to de-identify data. Following the interviews, interviewers wrote observational memos to capture immediate thoughts and perceptions of the interview [25] and updated a participant log with demographic information of the participant pool. Audio recordings were professionally transcribed. A preliminary codebook was created by AR, MY, JE, and LO based on interview domains. Transcripts of the first 5 participants were then reviewed and coded by AR and MY. To establish consistency, AR and MY compared codes and developed rules to guide future coding. New codes were added through line-by-line coding and thematic grouping until thematic saturation was determined to have been achieved. Iterative coding of the remaining transcripts was then conducted by XX and YY. Data were analyzed using MAXQDA software. The COnsolidated criteria for REporting Qualitative research (COREQ) Checklist was consulted to confirm comprehensiveness of the present study [26].

## Results

Twenty individuals participated in qualitative interviews, of whom 13 utilized formal harm reduction services through a local harm reduction organization. The remaining 7 interviewees were PWUD who did not utilize formal harm reduction services and who were referred to interview by their peers. The mean age of interviewees was 38 years. Overall, nine were female, and 19 identified as White and one as Black. All interviewees reported completing high school and two of them completed an associate degree or trade school program. Polydrug use—the concurrent use of multiple drugs—was common among interviewees. Eleven reported that their preferred drug was methamphetamine, while five preferred heroin, and seven preferred fentanyl. Among those who reported preferences for both methamphetamine and opioids, the drugs were reported as being used separately rather than combined.

We divided participant insights into the following four themes: (1) the impact of COVID-19 on participants’ quality of life and drug use, (2) attitudes around COVID-19 and vaccination efforts, (3) attitudes toward harm reduction-based healthcare, and (4) secondary distribution of harm reduction services and supplies. Pseudonyms are used throughout.

### Impacts of COVID-19

Coupled with a rural backdrop characterized by unique socioeconomic challenges [3], the pandemic presented financial challenges to many participants. Furthermore, the pandemic, public health measures such as social distancing, and concerns over financial and housing insecurity had serious impacts on participants’ mental health. Several also noted changes to drug quality and availability throughout the pandemic and consequent effects on drug use and risk behaviors.

### Socioeconomic impacts

Despite most interviewees receiving some financial support throughout the pandemic including government-issued stimulus checks, several expressed that these were insufficient. One example was Karen, a 40-year-old woman who lost her job, then lost access to reliable transportation—a particularly acute need in rural areas—because of the pandemic. Of the overlapping barriers to financial stability, she said,

I had a job before COVID hit and then, of course I lost my job because everybody lost – it’s not everybody -- lost their job. And now it’s just harder to even, yeah there's jobs out there, but I don't have a car now, so it's a lot harder to get out there and get a job and stuff like that, because of the whole car situation.

Two other interviewees, Rachel (a 38-year-old woman) and Rick (a 35-year-old man) discussed the impact of the pandemic on their ability to perform their job duties, as both worked with older adults who were not comfortable receiving in-person services during the pandemic. Since both were self-employed, there were no accessible supports in place to mitigate this loss of income. Rachel emphasizes that,

It sucks. I clean houses to make money. I lost all of them because they’re elderly people I clean for. They’re afraid of catching it, especially with me having all these kids.

A mother of six, she discussed the multiplying effects of this income loss. Without her income, Rachel was unable to pay for gas and internet services—barriers which led to no heated water in her home and her 14-year-old daughter being unable to complete her schoolwork virtually. Rachel further said, *“It's been a mess. Oh, it's been a mess, but I can't access and I have no internet and I can't go anywhere. It's been a mess because I don't know who can help me, who can't help me.”*

### Mental health impacts

However, for many interviewees, the impacts of the pandemic on quality of life were significant. Sarah, Rachel, and Josh, a 29-year-old man, described their mental health experiences over the last year as “*an emotional roller coaster*,” “*miserable*,” and “*broken*,” respectively. Mindy, a 45-year-old woman, dealt with the stress of a volatile break-up during the pandemic and subsequent mental health challenges. She said,

And then with COVID, when [my ex] couldn’t work, that really put a financial strain on a lot of things. And like we were having problems in our relationship and -- well, needless to say it didn’t work out and we just ended up splitting up. Now I'm just, basically to be honest with you, homeless and I’ve been staying with a friend. He just told me today that I had to get out. I really don't have nowhere to go… it’s definitely not easy. I’m depressed real bad.

For some participants, the pandemic caused substantial trauma and exacerbated other preexisting mental health stressors. Others were less psychologically impacted. Among those who did not feel that the pandemic notably impacted their daily life was Tom, a 41-year-old man. Tom described his resilience in the face of isolation due to experiences with incarceration, saying,

No, like isolation doesn't bother me a whole lot because I've been incarcerated, all together it's been about nine years total…so isolation is not a problem to me. I can keep myself occupied.

The responses to interview questions around wellbeing during COVID-19 were quite heterogeneous, reflecting the diversity of experiences and effects of the pandemic among this community. However, issues of housing stability and homelessness, coupled with domestic disputes, were major mental health stressors for most interviewees.

### Drug use impacts

Several interviewees commented on changes in the drug market during the COVID-19 pandemic. Many remarked that drug prices increased, while quality decreased. Josh explains:

Prices of drugs been just straight-up bonk[ers]. When you're told a price and you're told what you're going to get and then they show up and then they hand you something and its different…Quality and shit, quality’s went down.

Gary, a 30-year-old white man, similarly felt that there were reliability issues with the drug supply. He notes: “Yeah. They’re not the greatest. You never know if there's actually going to be what it's supposed to be or anything.”

Many remarked on the increased presence of fentanyl in their drug supply. Sarah remarked on the quality of methamphetamine,

We had a very high shortage on it [methamphetamine during the pandemic], and it was in very high demand. And then people started cutting it… It started coming back around, but then it started coming back around with traces of fentanyl.

Emily, a 30-year-old white woman whose drug of choice was methamphetamine, discussed the challenges of avoiding inadvertent opioid use in the context of a contaminated drug supply. She reported that she used fentanyl test strips each time she injected drugs, adding,

I used to be a heroin addict and that was like five years ago, and I still don't do that and I just do speed but… Some of it, you know, was positive for fentanyl and I don't want to do any type of opiate, no matter how small the amount.

Samantha, a 54-year-old white woman, remarked that the increased prevalence of fentanyl in her community was leading to increased overdose rates. She said,

I think what came out the most was the fentanyl, is it used to be actual heroin and now it's just fentanyl, these little beans. I didn't even know what a bean was until about a year ago, you know, it’s like a little capsule and it's got fentanyl in it and a lot of people ODed on that stuff because they thought it was heroin. They didn't know it was still an opioid. They didn't know. But a lot of people -- I lost a lot of people period.

### COVID-19 and vaccination efforts

Stigmatizing or otherwise uncomfortable experiences with healthcare professionals were cited by many interviewees as reasons to avoid interaction with the healthcare system. Since these experiences typically predated the pandemic, they informed interviewees’ attitudes toward COVID-19, especially with respect to vaccination efforts and mistrust of healthcare providers.

#### Previous healthcare experiences

Many participants described situations that drove them away from accessing healthcare, whether as a result of judgement, fear of criminal-legal repercussions, or a sense that their concerns were invalidated by providers. Sam, a 36-year-old white man, described concerns that the hospital was involved with the criminal-legal system. He said, *“People kind of think that they're going to get in trouble [with law enforcement] if they go to the hospital.”*

Experiences with both enacted and anticipated stigma in healthcare settings presented significant barriers to accessing healthcare among this population. When asked about the healthcare she received, Marissa, a 29-year-old white woman, said, *“The nurses made me feel very uncomfortable. Like, they were judging.”*

Karen (40, W) also described a particularly salient experience with a lab technician that kept her from returning to see her provider, with whom she had planned to work on reducing her drug use. She said,

So I went to my doctor. And then when I went to the lab to get my work in, they looked at my arm. I mean, she just, the lab lady just judged me. So, I didn't go back for help… whenever she says, “Oh, well, I'm sure you're not used to a needle this small” …you know, stupid, stupid stuff like that, how are you supposed to feel about that?

#### Attitudes around COVID-19 risk

Among interviewees, the health impacts of COVID-19 were often described as less significant than the impacts on other aspects of life, such as financial stability and drug quality. Rob, a 55-year-old white man reflected on his doubts around the severity of COVID-19. He said,

Well, it’s just another flu virus…They haven’t cured the flu in how many years and they’re gonna cure this overnight? No. That’s all. I think it’s all government. See how far they can push us.

Rick (35, male) expressed a lack of concern over the severity of the pandemic and indifference towards public health measures such as mask-wearing. He said,

I really don't consider it or think about it because if it is supposed to kill me, it’s going to get me. If it was meant to kill me, it's going to get me regardless.

Josh, one of the few interviewees who expressed concern over the spread of COVID-19, stated that he did not feel comfortable around many of his friends as they did not uphold public health strategies for preventing infection. He said,

It's made pretty hard to even trust…last year or so I started becoming a recluse. I started making it a point to not even let my friends into the house because…it was unknown whether or not they were vaccinated. One of which I guess I had pretty good reason to because I had slipped up one time and a girl was inside the house and was like, *I was notified earlier today that I should be*, she mentioned something about *should be quarantining*. And this was literally -- this was before we got vaxxed…She's in the house nonchalantly.

When asked about their primary source of information about COVID-19, most individuals replied that they found information on the internet or learned about the virus and vaccine efforts through word-of-mouth. Rob (55, M) said: *“I live on Google, studying up on all this.” The information they gathered through these searches informed their perception of COVID-19 and vaccination efforts, discussed below.*

#### Attitudes toward COVID-19 vaccination

Despite public vaccine rollout beginning in early 2021, only 4 out of 20 interviewees (20%) had received a vaccination against COVID-19 at the time of their interviews. Among several interviewees, a distrust of the healthcare system and government were predominant reasons for declining vaccination.

Several interviewees did not believe that the evidence for vaccines was strong enough to warrant vaccination. Rachel compared the pace of vaccine production for COVID-19 with other public health crises, saying, *“Nope. We've had AIDS for how many years and don't have a cure. COVID just came out and they automatically have a cure for that, a vaccination.”*

Rick also endorsed uncertainty over the long-term effects of the vaccine, adding, *“Give it 10 years and we'll see people growing extra limbs or whatever from it, or they're having deformed babies or babies with disabilities and stuff like that, then we’ll see.”*

Several interviewees also cited concerns around the contents of the vaccine, largely in the context of information they had garnered via word-of-mouth or posts on social media. Alice said,

So, the COVID vaccine, I figure I should probably do research on the new vaccines…they had Caucasian male fetus lung tissue in it in the first original vaccine and I didn't see a purpose in that. The 12-week-old fetus lung tissue. Why is that in there?

Sarah echoed some of these concerns, and added that she worried about potential adverse effects of the vaccine. She said,

I'm not putting baby organs inside me, I'm sorry. They grow inside me…plus, a nurse was the very first person to get a COVID vaccination, 20 minutes after taking her first shot, she died. Hell, no. And there's still people dying from it. And you got to take more than one, sorry. If anybody's going to poke me, it's going to be my damn self. Sorry.

Among those who were vaccinated, information from trusted sources and motivation to protect others provided much of the impetus for vaccination and helped to reduce distrust. Emily, a 30-year-old white woman, said,

First I was really sketched out…I’m one of those paranoid people. But one of my good friend's husband works for the NSA or has like contracts with NSA, like super smart, and then he said it was like he got it and he just kind of talked me into it…I think it's a very small thing for people to do to in a big scheme of things, possibly saves other people's lives.

Greg, a 30-year-old white man whose father was immunosuppressed after a liver transplant, said,

I was in a halfway house, and I had to get the vaccine to be able to have any movement whatsoever. But I'm glad I got it. I would have gotten it because of my dad anyway so. I can't really make such a big deal about, you know, being super concerned about what's in it and all that. I mean, I shoot dope, you know what I mean.

When asked about his rationale for being vaccinated, Jim, a 51-year-old white man, said, “So, I can have an in-person visit with my friend…in prison. So, there’ll be no screen in front of us.”

Given the appearance of new COVID-19 variants and particular susceptibility of this population to adverse effects of COVID-19, these attitudes toward vaccination efforts were particularly salient. Participants often expressed distrust of healthcare providers, vaccine manufacturers, and government officials. In contrast, many interviewees expressed greater trust in and comfort around harm reduction providers, as well as receptivity to healthcare services offered in a harm reduction context. These findings are discussed below.

### Community-based harm reduction and healthcare

Participants responded to interview items regarding barriers and facilitators to accessing harm reduction services that they experienced during the COVID-19 pandemic. Interviewees also discussed their openness to potential healthcare services that could be offered through or in collaboration with the local peer-operated harm reduction organization in the future.

#### Attitudes toward harm reduction providers

While interviewees often associated healthcare experiences with stigma and discrimination, many saw their local harm reduction organization as a trusted source of care and support. One feature of the local harm reduction organization that resonated with interviewees was the focus placed on convenience and accessibility. By delivering supplies in addition to operating at fixed sites and driving participants to service providers when possible, the organization addressed transportation concerns common among rural PWUD. Samantha, who did not have access to independent means of transportation, expressed gratitude for these efforts. When asked if there were any barriers to her accessing services, she said,

Not at all because [the harm reduction provider] always comes and gets me and he's really good about doing that. He puts himself out there, so he really does. I enjoy it, like today he came and got me and did all this.

Rachel, who was also unable to access transportation during the pandemic, added that she would not have been able to obtain supplies like sterile syringes and naloxone without the harm reduction organization’s home visits. She said,

If [the harm reduction organization] wouldn't come here then I wouldn't be able to [access services]. I like it that they deliver... Or you guys come in here and you can pick us up and take us to wherever you're at.

Furthermore, the harm reduction organization provided supplies discreetly to PWUD who may not want attention drawn to themselves. According to Wayne, a 47-year-old Black man, supply delivery was *“sweet and discreet.”* Emily discussed the benefit of having a harm reduction organization to provide supplies for safer drug use among those who may feel stigmatized in other settings, saying,

Some people are too embarrassed to go buy new needles and they’ll just use the same packs that they've got for months or end up sharing. And there's a lot of diseases and stuff like that, and people don't really care when they're in it bad. We don't think of it. And it just really does make it easier for us… they're bringing us supplies.

Interviewees also noted that their harm reduction provider placed an emphasis on knowing PWUD as people first, rather than immediately attaching a label or stigma to them. Greg summarized this impression, saying,

I'm 100% comfortable with [the harm reduction provider]. He's been nothing but a saint to me and [interviewee’s partner]. And I just think like, I have so much respect for him for bringing a harm reduction service to our area, which we had absolutely nothing to speak of. And like the way he went about it, and just doing it himself, like, I can't say enough about how much I respect that.

Alice expressed feelings of comfort with members of the harm reduction organization. She said,

All the staff, everybody that I've ever been interviewed by, or talked to on the phone has always been very comfortable, very welcoming. No one's ever made me feel unwelcome.

Spencer, a 39-year-old white man, added that the humanistic element of the care provided by the harm reduction organization was particularly meaningful to him. He said,

I mean, besides all the help [the harm reduction provider] gives…just in general, talking to him as a human being and a friend, I mean, he's a really good guy. So, I mean, it's not all just based on the services he provides, it's, you know, we're -- there's more to it than just that, I guess, so it's kind of nice.

#### Enthusiasm for harm reduction organization-based healthcare

In addition to expressing support for their local harm reduction organization, many interviewees were enthusiastic about the prospect of accessing healthcare services through the organization [27]. Interviewees were asked about which medical services they would be interested in receiving should they be offered through the local harm reduction organization in the future. For Samantha, Wayne, and Alice, respectively, dental care, sexually transmitted infection testing, and treatment for chronic conditions such as hypertension were exciting future possibilities. In Samantha’s words,

I know so many people that need a dentist, or just you know, help getting services or something…[Medicaid] is not for teeth. So, a lot of people have a lot of teeth problems and that's painful.

Sam (36, M) and Rachel (38, W) also expressed interest in wound care being offered through the harm reduction organization, largely because of the stigma associated with drug use in wound care settings. In Sam’s words,

The wound care…Because that would be one of the main things that people get- abscesses and stuff like that.

Rachel agreed, and discussed a friend of hers who could benefit from such offerings. She said,

The wound care would be good. An acquaintance, he goes to wound care because from his knees down he shoots up in his legs and he got ahold of that Krokodil shit…He's embarrassed to go to wound care because they're judgmental knowing he shoots up.

Josh, one of the four interviewees who had received a COVID-19 vaccination at the time of interviewing, was in support of the local harm reduction agency offering COVID-19 vaccinations to PWUD in a trusted environment. He said, *“That would kind of be neat to see…that would be cool.”*

Several interviewees who had not yet been vaccinated stated that, if they were required to receive the COVID vaccine, they would be comfortable receiving it in the welcoming space of the harm reduction organization with trusted professionals nearby. When Samantha was asked where she would be most comfortable receiving the vaccine, she replied, *“[With] this guy. I like [the harm reduction provider]. He’s cool, he’s awesome.”* Rick expressed a similar sentiment when asked the same question. He said, *“Probably in a place like [the harm reduction organization’s mobile van unit] or at home.”*

Other potential forms of healthcare that could be offered in a harm reduction setting are hepatitis C treatment and testing. When he was asked about several healthcare services, Tom said,

They need all that stuff. I can’t say that personally somebody that I know that has HIV or AIDS, but I know several people that have Hep A, Hep B, Hep C, Hep-15, whatever.

Josh agreed and elaborated on the difficulties of obtaining HCV treatment as the system stands. He said,

Treatment for Hepatitis C sounds cool. You have to literally like go to the specialist, you have to get referred to them…and you have to have your doctor refer you to them, which is ridiculous.

Alice added that she would be more likely to utilize healthcare services including treatment for hepatitis if they were offered in this setting. She said,

I would definitely utilize, I would get more medical help myself, which is hard for me to even do, but I would probably actually might even consider doing hepatitis treatment again, consider it maybe but I might consider doing it. I went through interferon, it was horrible.

Beyond access to healthcare services via the harm reduction organization, 19 of the 20 interviewees agreed that offering a computer or tablet in the harm reduction organization’s mobile unit for people to engage in telehealth appointments would be valuable. Samantha summarized many interviewees’ sentiments, discussing the lack of access currently facing many PWUD with respect to telehealth. She said,

Well, yes because some of us don't have computers at home. I don't have a computer…I mean, you can put it in a little TV monitor in there and get wi-fi hotspot. Yeah. That makes absolute sense to use it for the whole program really, because, really, it's overall health, so makes sense.

In discussing the possibility of harm reduction-based healthcare services, many interviewees expressed excitement at the prospect of increased accessibility. Wayne summarized the widespread need for interventions such as these in promoting community health. In his words,

I guess one person's needs is someone else's need too. So eventually, you're going to have to see that like, well, maybe they need that.

Despite the widespread enthusiasm for the local harm reduction provider and possibilities for accessible healthcare, many interviewees expressed wishes that a greater number of community members would utilize harm reduction services. Various reasons for the reluctance to do so included stigmatization of drug use, fear that there might be criminal-legal repercussions, and unawareness of the presence of the harm reduction organization. One means for addressing this gap in service delivery is through peer networks of PWUD. Among interviewees who did access harm reduction services and supplies from the local harm reduction organization, 12 out of 13 indicated that they engaged in secondary distribution, the process by which one PWUD obtains harm reduction supplies to distribute to other PWUD [11], or that they intended to do so in the future. Thoughts from these interviewees, as well as interviewees who access informal harm reduction services through their peers, are discussed below.

### Secondary distribution

“When I quit using needles, I'm still going to get supplies, hopefully from [the harm reduction provider] if [they] will do it for me, because I supply others…. Every time [the harm reduction provider] would come see me, [they] would bring me double what [they were] supposed to, just because [they] found out that I was just giving.”—Sarah.

Many interviewees engaged in formal harm reduction services expressed a commitment to making sure that other community members were able to access services, even if they did not directly engage with a harm reduction provider. Alice, who had recently witnessed the overdose of a close friend, discussed her decision to become a secondary distributor of Narcan. She said,

Without [harm reduction] services, I'm sure more than just he would probably be gone because that was a preventable death. And so, yeah, and I do give out Narcan. And I teach them how to administer it… So, yes, I do provide the services that are provided for me. I readily keep them available for others.

Josh, a member of the community advisory board of the harm reduction organization, added that he provides sterile syringes to peers. He said,

I always come up to them with the unopened package of syringes, if I am going to give them an entire package. If I feel like I'm low (on syringes) or whatever…I will open it up in front of them and give some out of it, but I'll show them that it’s fresh, new, out of the package… I’m there for anyone.

When asked if she provided harm reduction supplies, Rachel said,

Narcan and clean needle kits…People come here daily wanting needles…They just pop in or call. “Do you have any clean needles we can get?” “Yeah come and get some.” And I gave them a bag or two depending.

When asked about her rationale for distributing supplies, she added,

I caught Hep C from using a dirty needle and the lady knew she had Hep C and didn’t even say anything to us. That's how me and [interviewee’s partner] caught it. If I can give somebody clean needles so they do not catch it like I did, I will give them clean needles.

Wayne, one of the interviewees who did not report participating in secondary distribution in the past, made a commitment to do so in the future. When asked if he had ever provided sterile supplies to his peers, he said: “No, I haven't but I will. I have a couple friends who come over to do heroin.”

Among those who did not access formal harm reduction services through the local provider, all reported previous receipt of supplies via secondary distribution. Gary, who was connected with the local harm reduction provider for future services at the time of his interview, said of his previous receipt of harm reduction supplies,

I mean, for two-and-a-half years I've always made sure I've had or I've gotten naloxone from somebody in general that’s got it from the clean needle exchange I would say… Word of mouth [about formal services] finally spread to us I guess.

Cathy, a 33-year-old white woman, reported receipt of sterile syringes via secondary distribution. She said,

My friend… never used the needles or anything like that. So, all the ones extra that he had, he ended up giving to us.

Mindy also accessed sterile syringes from peers, citing the prohibitive cost of accessing syringes through the local pharmacy. She said,

Yes, you can also go to like pharmacies to get them. Sometimes if you don't have the money to do that effectively…[secondary distribution] could be very helpful. Because yeah, everything has got to be sterile for sure.

When asked about having supplies delivered from the local harm reduction organization in the future, Mindy added,

Yeah, I’d be good with that because they brought it right to [Karen’s] house… you've opened my eyes to a lot of stuff, because I didn't know about that. And I'm definitely going to use that other thing - the mobile [van unit].

Cathy summarized the attitude of several interviewees who wished that the local harm reduction organization had more funding and ability to conduct outreach so that services were available to more community members. She said,

I just think to be able to branch out more to be able to get more employees or more volunteers that were able to hit more area and to be able to contact more people and get more information across, I guess… Yeah, there really isn't anything like him here. I've heard of it, different places, Canada or different hotspots, cities, I guess, in the United States that offer even places to, I guess, go use drugs in a safe environment…but there's just not things around here, I guess more because it's a rural area. It's just not offered to us down here.

Whatever an individual’s reasons for not engaging in formal local harm reduction services, secondary distributors make safe, sterile supplies and community support much more accessible to their peers. In Alice’s words,

New cases of STDs and HIV and all that stuff, they got to be going down by even a little- even one number is a big deal. And I know that just for me alone, I feel like I've gotten to touch that number somehow and just by my house, even just the one person, me alone, just giving them new [syringes], encourage them not [use old syringes]. Don't do that. Don't do that- here, take what you need.

## Discussion

Findings from this qualitative investigation of PWUD in rural southern Illinois indicate that the impacts of the COVID-19 pandemic on daily life and drug-related risk factors intersect with stigmatization and negative medical experiences to create a number of overlapping barriers to service access and vaccine uptake. Though participants cited a variety of obstacles to achieving quality healthcare, several also shared their ideas for surmounting these challenges. Ideas for future services supported by interviewees included offering healthcare services (e.g., wound care, hepatitis C treatment and testing) in concert with harm reduction services, facilitating telehealth in the harm reduction organization’s mobile unit for remote doctors’ visits, and increasing the involvement of community members and peers in service delivery to PWUD as well as in advisory bodies for harm reduction organizations.

Our study adds to the limited body of research that qualitatively explores the experiences of PWUD with healthcare and harm reduction services during the COVID-19 pandemic. Previous works have explored the impact of the pandemic on mental health and overdose risk [28], adaptations to sterile syringe programs [29], and overdose response during the pandemic [30]. Our study contributes to this literature by investigating the experiences of PWUD in seeking out health and harm reduction services while navigating a rural risk environment, barriers to healthcare, and a pandemic with impacts on drug quality, availability, and risk behaviors as well as financial, social, and mental wellbeing.

Our study supports previous work around the disproportionate impact of the COVID-19 pandemic on PWUD. Results support large-scale studies of PWUD indicating that access to financial resources and downstream effects such as housing insecurity have been a significant detriment to the lives of many PWUD. Results also support studies showing that the pandemic has negatively impacted mental wellbeing [28]. Participants also corroborated concerns discussed in past studies regarding decreased drug availability, increased drug cost, increased contamination of drugs with other substances such as fentanyl and fentanyl analogs, and perceived increases in overdose rates within their community [31]. These concerns highlight the need for interventions related to the impact of COVID-19 specifically tailored to meet the unique needs of PWUD.

Our findings also reinforce current literature on the relationship between PWUD and the healthcare system as it stands. Our work supports past studies of PWUD in rural settings that found stigma and inappropriate treatment of medical problems significant barriers to accessing healthcare services [32]. This reluctance to seek healthcare is particularly concerning during the COVID-19 pandemic, as untreated and unvaccinated PWUD are likely to face elevated morbidity rates from both COVID-19 and substance use [29]. Furthermore, results from this study add to the list of concerns around access to credible and actionable information among PWUD when it comes to COVID-19. Many PWUD lack access to a regular healthcare provider whom they trust and receive most of their information about COVID-19 through word of mouth or via the internet. This lack of information or inaccurate information about COVID-19 may lead to decreased vaccine uptake and further exacerbate COVID-19 health risks facing this community, especially in the wake of highly infectious variants [33].

We explored the ways in which PWUD collectively promote the health of their community through engagement with harm reduction services, secondary distribution, peer support, and enthusiasm for instituting community organization-based healthcare services. We also explored the ways in which PWUD continue to be marginalized and rejected from societal structures, such as through stigmatization by healthcare professionals, that may lead to hesitancy to access healthcare or receive COVID-19 vaccinations in the future. Utilizing suggestions from interviewees, we suggest several possible interventions for addressing the issues discussed above.

First, integrating a wider range of healthcare services into the existing local harm reduction infrastructure would enable PWUD to access important services in an atmosphere viewed as less judgmental and more accessible than typical healthcare settings. Consistent evidence pointing to the efficacy of harm reduction as well as the breadth of health disparities among PWUD supports calls for such integration [34–36]. If implemented, a variety of services could be offered including wound care, hepatitis C testing and treatment, and COVID-19 information dissemination, testing, and vaccination. Such services could be offered through providers affiliated with the local harm reduction agency via their mobile van units that currently distribute harm reduction supplies to clients at multiple sites each week. By incorporating healthcare services into the current harm reduction model, PWUD and activists can address health needs of community members at a grassroots level while continuing to fight for more widespread, institutional reform that makes the healthcare system at large a safer place for PWUD.

Along with general expansion of accessible healthcare, harm reduction providers have the potential to become stewards of up-to-date information about COVID-19 and likely future infectious diseases or other emerging health risks for PWUD who may not have access to or trust in healthcare institutions’ recommendations around pandemic response and vaccination. Historic disenfranchisement of PWUD and institutional untrustworthiness warrants their skepticism of institutions such as healthcare, but leaves them with fewer credible sources of information about the pandemic. Many interviewees’ concerns regarding vaccination revolved around fears that vaccine development was rushed and that side effects may occur. Harm reduction providers and allied health professionals are well-positioned to engage PWUD in conversation around these concerns and provide accurate information in a non-judgmental space where PWUD are encouraged to ask questions and learn more about vaccines.

Second, for those health concerns that cannot be addressed in the harm reduction organization’s space, PWUD living in rural areas may benefit from telemedicine visits conducted with computer stations available in the organization’s mobile units. Even as in-person visits become the norm once more, technology’s involvement in delivering patient care will likely persist [37], although the benefits will likely not extend equitably among patients. For patients who use drugs, access to providers via telemedicine may be impacted by inconsistent cellphone access, lack of familiarity with telehealth services, privacy concerns, and limited broadband particularly in rural areas, compounding upon other existing barriers to accessing healthcare among this population [38]. By implementing technology stations at trusted local harm reduction organizations, an idea endorsed by 19 of the 20 participants in this study, PWUD may be able to access medical care in a COVID-safe and comfortable way. Additionally, increasing the availability of accessible telehealth services would help to address geographic and transportation barriers to accessing healthcare in rural settings, and mitigate some of the widening disparities caused by disproportionate uptake of telehealth services in urban compared to rural regions. [39]

Finally, the importance of secondary distribution of harm reduction services and supplies among this study’s participants highlights the need for continued engagement of PWUD in the development and implementation of future harm reduction interventions. This involvement may help to maximize the network of individuals reached and number of services and supplies delivered. The barriers cited by PWUD to accessing care and the response of community members in promoting access highlight the mobilization of resources among peer networks in response to inadequate institutional support for PWUD health. This response is crucial as secondary distribution recipients may be younger, less experienced, and at higher risk of negative health consequences related to drug use [40]. Research suggests that peer support may be an effective strategy for fostering education, community, and safer drug use among those endangered by such health disparities [41, 42] and several participants in this study made clear that their close relationship with peers empowered them to distribute or receive important health services. By integrating these individuals into service delivery, peer education, and administrative structures such as community advisory boards, a wider community of PWUD—especially those at higher risk—may be served, and gaps in trust between community members and providers may begin to be bridged.

Our study has several limitations. These include the lack of racial diversity among our participants, though this reflects the area’s population, and the limited geographic distribution of the study sample, and the small sample size. Results may not be generalizable to all PWUD in the study region or beyond. Furthermore, our study sought to assess the attitudes and experiences of PWUD during the pandemic, and thus we did not interview any other stakeholders. Finally, interviews were conducted in the summer and fall of 2021, a full year after the COVID-19 pandemic reached the United States and prior to the Omicron variant becoming the dominant strain of COVID-19. It is difficult, therefore, to judge whether participants’ experiences have varied over the course of the pandemic and whether responses would have been different a year ago.

## Conclusion

Distrust of healthcare institutions and lack of credible information about COVID-19 impact the ways in which PWUD perceive the severity of COVID-19 and the potential harms of vaccination. By incorporating community-delivered healthcare services and credible COVID-19 information within an existing local harm reduction framework, as well as integrating peer support and community advising, healthcare and harm reduction providers may have a greater ability to repair trust and address unique health disparities facing PWUD.

## Data Availability

The data that support the findings of this study are available on request from the corresponding author [AR]. The interview files are not publicly available due to them containing information that could compromise research participant privacy/consent.

## Abbreviations

PWUD: People who use drugs
MOUD: Medications for opioid use disorder
ETHIC: Ending Transmission of HIV, Hepatitis C, Sexually Transmitted Infectious and Overdose in rural communities of people who inject drugs
CFIR: Consolidated Framework for Implementation Research
COREQ: COnsolidated criteria for REporting Qualitative research
